# Biodistribution and Clearance of Stable Superparamagnetic Maghemite Iron Oxide Nanoparticles in Mice Following Intraperitoneal Administration

**DOI:** 10.3390/ijms19010205

**Published:** 2018-01-10

**Authors:** Binh T. T. Pham, Emily K. Colvin, Nguyen T. H. Pham, Byung J. Kim, Emily S. Fuller, Elizabeth A. Moon, Raphael Barbey, Samuel Yuen, Barry H. Rickman, Nicole S. Bryce, Stephanie Bickley, Marcel Tanudji, Stephen K. Jones, Viive M. Howell, Brian S. Hawkett

**Affiliations:** 1 Key Centre for Polymers and Colloids, School of Chemistry, University of Sydney, Sydney, NSW 2006, Australia; phamthuybinh@yahoo.com (B.T.T.P.); nguyen.pham@sydney.edu.au (N.T.H.P.); Bkim5239@gmail.com (B.J.K.); raphael.barbey@a3.epfl.ch (R.B.); nicole.bryce@unsw.edu.au (N.S.B.); 2 Bill Walsh Translational Cancer Research Laboratory, Kolling Institute, Royal North Shore Hospital, Sydney, NSW 2065, Australia; emily.colvin@sydney.edu.au (E.K.C.); emily.fuller@sydney.edu.au (E.S.F.); e.millar@sydney.edu.au (E.A.M.); samuel.yuen@sydney.edu.au (S.Y.); 3 Sydney Medical School—Northern, University of Sydney, Sydney, NSW 2006, Australia; 4 Sydney School of Veterinary Science, University of Sydney Teaching Hospital Camden, Camden, NSW 2570, Australia; barry.rickman@gmail.com; 5 Sirtex Medical Limited, North Sydney, NSW 2060, Australia; s_rozeleur@hotmail.com (S.B.); mtanudji@sirtex.com (M.T.); skjones314@gmail.com (S.K.J.)

**Keywords:** superparamagnetic maghemite iron oxide nanoparticle (SPION), steric stabilization, intraperitoneal biodistribution, macrophage clearance

## Abstract

Nanomedicine is an emerging field with great potential in disease theranostics. We generated sterically stabilized superparamagnetic iron oxide nanoparticles (s-SPIONs) with average core diameters of 10 and 25 nm and determined the in vivo biodistribution and clearance profiles. Healthy nude mice underwent an intraperitoneal injection of these s-SPIONs at a dose of 90 mg Fe/kg body weight. Tissue iron biodistribution was monitored by atomic absorption spectroscopy and Prussian blue staining. Histopathological examination was performed to assess tissue toxicity. The 10 nm s-SPIONs resulted in higher tissue-iron levels, whereas the 25 nm s-SPIONs peaked earlier and cleared faster. Increased iron levels were detected in all organs and body fluids tested except for the brain, with notable increases in the liver, spleen, and the omentum. The tissue-iron returned to control or near control levels within 7 days post-injection, except in the omentum, which had the largest and most variable accumulation of s-SPIONs. No obvious tissue changes were noted although an influx of macrophages was observed in several tissues suggesting their involvement in s-SPION sequestration and clearance. These results demonstrate that the s-SPIONs do not degrade or aggregate in vivo and intraperitoneal administration is well tolerated, with a broad and transient biodistribution. In an ovarian tumor model, s-SPIONs were shown to accumulate in the tumors, highlighting their potential use as a chemotherapy delivery agent.

## 1. Introduction

Over the last two decades there has been an increase in the use of magnetic particles and magnetic microspheres including iron oxide nanoparticles (IONPs) and superparamagnetic iron oxide nanoparticles (SPIONs) for bio-medical applications such as imaging, diagnostics, therapies and theranostics, but their suitability for these applications ultimately depends on their biodistribution and toxicity profiles [1,2,3,4,5,6,7,8,9,10,11,12,13,14,15,16,17,18,19,20]. The two main administration routes for IONPs into the body are intravenous (IV) injection and intraperitoneal (IP) injection. While IP injection offers a longer plasma half-life of particles since it takes longer for the particles to reach circulation, a limited number of studies have been reported, and IV injection remains the most common clinical administration route [8,19,21,22,23,24,25,26]. Biodistribution studies have found that IONPs are localized primarily to the liver and spleen for clearance via mononuclear phagocytes [3,4,7,10,11,13,27,28,29,30,31]. For example, Feridex^®^ (a colloid of SPIONs associated with sugar-based polymers) received U.S. Food and Drug Administration approval in 1996 for targeting liver and spleen lesions, but was discontinued in 2008 due to severe side effects [32].

The majority of IONPs have significant side effects due to their prolonged retention in the body for time periods up to 11 months [7,33]. Several biodistribution studies performed with Feraheme™ (IONPs surrounded by a carbohydrate coat) demonstrated retention times longer than 7 days in mice, with IONPs still detectable in the liver and spleen of the treated mice at this point in time [10,12,13,34]. Ma and co-workers [19] reported that for IP injection of magnetic 35 nm diameter positively charged Fe_3_O_4_ nanoparticles from Sigma-Aldrich (Castle Hill, Australia), the safe upper limit was 5 mg/kg administered daily to mice for one week. This equates to a total injection of 35 mg/kg body weight per mouse. Above this concentration, they observed significant toxicity to both the liver and kidneys with increases in reactive oxygen species (ROS) and changes in the tissue histology. Since analysis of the iron concentration was not performed, there was no direct link between the presence of nanoparticles and tissue toxicity. Work by Proden and co-workers [8] found no significant changes in the morphology of the liver, spleen, or lungs of Brown Norway rats 48 h after IP injection of 10 nM IONPs up to 3.7 mL/kg (equivalent to approximately 3 mg/kg). However, the kidneys showed pronounced distortions in the tubular cell architecture at the highest dose.

The work by Proden and co-workers [8], discussed above, reported that the long-term accumulation of IONPs in the body could be an advantage for imaging and therapeutic applications. It is also important to consider the long-term effects of IONPs being deposited in the liver and other organs as a result of particle aggregation. This could lead to inflammation and fibrosis of those organs. As the majority of reported biodistribution studies do not quantify iron content over time in the tissues of interest, analysis of the pharmacokinetics and the toxicity profiles of IONPs remain difficult to interpret. It is also hard to distinguish whether the cytotoxic nature of IONPs is due to colloidal instability, owing to the poor stabilization of the particles, or the degradation of the particle core and coating. When IONPs are not stabilized appropriately, the particles may aggregate, impeding or slowing the clearance of the IONPs from the injection point or from the first sequestering tissues. This leads to long-term exposure, which may result in toxicity. For IONPs, a possible cause of toxicity may be the use of magnetite iron oxide based particles, as the Fe^2+^ ions in Fe_3_O_4_ cores are not chemically stable and are susceptible to oxidation to form the more stable Fe^3+^ ions. A significant quantity of free iron cations form free radicals, especially ROS, which damage intracellular organelles and can lead to adverse long-term side effects. In addition, considering that the iron content of a number of commercially available IONPs (Nanoprobes, MagForce, Micromod, NanoMaterials Technology, Feraheme) is low (10–30 wt. % of dry mass), the composition of the particle coating may also play a role in determining the cytotoxicity of IONPs.

Our customized γ-Fe_2_O_3_ s-SPIONs have a very high iron content, up to 60 wt. % with a high T2 relaxivity of 368 and 953 s^−1^ mM^−1^ for 10 and 25 nm cores, respectively; the s-SPIONs are also stable in simulated physiological media and in the presence of fresh human red blood cells [35,36,37,38]. The in vitro co-administration of anti-cancer agents (chemotherapeutics) and these s-SPIONs led to a significant enhancement in the penetration of the anti-cancer agents, thereby inhibiting the proliferation and migration of cancer cells from tumor spheroids once the chemotherapy treatment was stopped [35]. In order to translate these promising in vitro findings to in vivo models it is critical to first determine the biodistribution and clearance of the s-SPIONs in healthy mice.

In this study, we generated customized γ-Fe_2_O_3_ s-SPIONs with reversible addition fragmentation chain transfer (RAFT) diblock copolymers and demonstrated their stability both at high salt concentrations and in physiological media. To better understand their potential for biomedical applications we then assessed the in vivo biodistribution and clearance of these s-SPIONs. Nude mice (BALB/c-*Foxn1^nu^*/Arc) were IP injected with 200 µL of Phosphate-Buffered Saline (PBS) with or without s-SPIONs at a dose of 90 mg Fe/kg body weight, which equates to 7.3 mg/kg in humans [39]. This dose is close to the clinical dose of iron oxide nanoparticles for the treatment of iron deficient anemia with the FDA approved IONPs (510 mg iron/injection for 60 kg body weight, i.e., 8.5 mg/kg) [27]. The iron concentration in the plasma and in major organs was determined quantitatively by atomic absorption spectroscopy (AAS), and histological Prussian blue staining was performed at 1, 4, 24, and 48 h as well as after 7 days post-injection. In addition, s-SPIONs were IP-injected into ovarian tumor-bearing mice to determine whether they localized to tumors in vivo.

## 2. Results

### 2.1. Generation and In Vitro Stabilization Studies of the SPIONS

#### 2.1.1. Macro-RAFT and Short Chain Diblock Stabilizers with Functionalized End Groups

In our earlier studies, in order to obtain macro-RAFT diblocks with an NH_2_ functionalized end group (*RAFT-MAEP*_10_-*AAm*_20_-*NH*_2_**), we modified the –COOH end group of the C_4_-RAFT agent on the *RAFT-MAEP*_10_-*AAm*_20_ diblock copolymers using standard *N*-hydroxysuccinimide/*N*-(3-dimethylaminopropyl)-*N*-ethylcarbodiimide hydrochloride (NHS/EDC) conjugation chemistry [35,38]. This process required an additional step after the diblock synthesis and was not reproducible in its control over the conjugation efficiency, i.e., the exact number of NH_2_ end groups on each particle. In this current study, by synthesizing *N*-Boc-ethylenediamine C_4_-RAFT from the C_4_-RAFT agent, no further modification was required after polymerization and macro-RAFT diblocks with a defined and homogenous functional end group were obtained. Characterization by Nuclear Magnetic Resonance (NMR) spectroscopy confirmed that the pure *N*-Boc-ethylenediamine C_4_-RAFT, the protected NH_2_ end functionalized RAFT agent, was synthesized successfully.

#### 2.1.2. Steric Stabilization Achieved through the Combination of SPIONs with RAFT Derived Diblocks with a Desired Ratio of Various Functional End Groups

**Figure 1:**
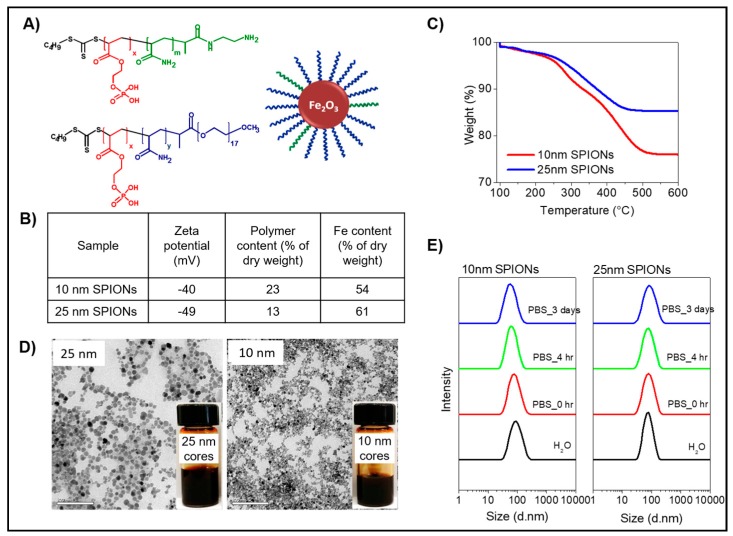
Characterization of superparamagnetic iron oxide nanoparticles (s-SPIONs). (**A**) Chemical structures of the steric stabilizers for SPIONs. Phosphate block (red) is the anchoring block to iron oxide surface; stabilizing blocks are a mixture of 95% MPEG (blue) and 5% NH_2_ end functionalized polyacrylamide (green) (*x* = 6; *y* = 0; and 40; *m* = 20 and 60 for small and big cores, respectively); (**B**) Properties of the s-SPIONs; (**C**) TGA showing the composition of the s-SPIONS; (**D**) TEM images of the 10 nm (**right**) and 25 nm cores (**left**) s-SPIONs; inset shows dispersion of SPIONs at 7 mg Fe/mL in PBS (scale bar = 200 nm); and (**E**) Dynamic light scattering measurements for particle size distribution and particle stability of 10 nm (**left**) and 25 nm (**right**) s-SPIONs dispersions with time in PBS.

The composition and size distribution of the 10 and 25 nm SPIONs stabilized with RAFT diblock copolymers with 95% methoxypolyethylene glycol (MPEG) and 5% NH_2_ functionalized end groups are shown in Figure 1. The negatively charged phosphate groups on the RAFT diblock copolymers are anchored to the surface of positively charged iron oxide cores, while the water soluble MPEG or polyacrylamide block extends into the dispersing aqueous phase (Figure 1A) [35,40]. The final zeta potential of the particles is strongly governed by the method of stabilization. We have previously shown that when the surface of the raw acidic ferrofluid core was fully coated and/or encapsulated by the polymer, the final zeta potential was determined by the charge of the polymer chain [40]. However, in this work the MPEG and polyacrylamide stabilizing blocks used are soluble in the continuous phase, the particle surface is bare except for the short anchoring blocks and therefore accessible to soluble molecules and/or (counter-) ions present in the aqueous phase. Thus, while the particles possess polyethylene glycol (PEG) and/or NH_2_-end functionalized polyacrylamide chains originating from the macro-RAFT diblocks as the main stabilizer (Figure 1B), the overall zeta potential of each SPION is negative due to the negative charge from the anchoring block [35].

The amount of polymer incorporated onto the final s-SPIONs was measured by thermogravimetric analysis (TGA). As shown in Figure 1C, the percentage of macro-RAFT diblocks incorporated onto the 10 nm s-SPIONs is greater than for the 25 nm s-SPIONs. The higher number of macro-RAFT molecules anchored at the interface of the smaller particles is expected due to their higher surface area compared to that of the larger particles. On the other hand, both s-SPIONs are rich in iron, accounting for 54% and 61% of the total dry weight for the 10 and 25 nm particles, respectively. Both the 10 and 25 nm core sterically stabilized particles demonstrate outstanding stability under physiological conditions (e.g., in PBS). Visual observation of the dispersions as captured in Figure 1D showed no aggregation or settling of particles over several weeks. Transmission electron microscopy (TEM) images of the s-SPIONs also demonstrated a narrow size distribution and well dispersed s-SPIONs when dried. The hydrodynamic diameter of the s-SPIONs was followed with time using a dynamic light scattering (DLS) particle sizer. As shown in Figure 1E, the 10 nm core s-SPIONs at the time of dispersion had a larger hydrodynamic diameter in water than in PBS. In addition, the peak of the hydrodynamic diameter of the 10 nm core s-SPIONs in PBS decreased with time; e.g., from 80–90 nm when freshly prepared in water compared with 40–50 nm after 3 days in PBS. These observations suggest that the hydrodynamic diameter of particles in suspension might be influenced by how well the stabilizers extend in the continuous phase. As the solubility of poly ethylene oxide (PEO) and PEG are known to depend on the solvents used [41,42], the hydrodynamic size of PEG stabilized particles is governed by the corresponding dispersing phase.

The PEG stabilized 10 nm s-SPIONs in water showed a larger hydrodynamic size due to the well described formation of crowded hydrogen bonding between the ether oxygen of the PEG chains and water molecules [41]. When the water based s-SPIONs were diluted with PBS for further in vitro investigation, a fresh sample prepared in PBS did not show significantly different hydrodynamic properties under light scattering measurements. However, over time the high concentration of cations gradually interrupted the hydrogen bonds and provided good shielding between the PEG and water molecules to keep the individual particles apart. This results in the decreased hydrodynamic size of the 10 nm s-SPIONs in PBS with time by light scattering measurements.

In contrast, the 25 nm s-SPIONs display a constant hydrodynamic diameter by DLS in both water and PBS over a 3 day period (Figure 1E). The short polyacrylamide block is needed together with the short PEG chain to provide adequate stabilization of the 25 nm SPIONs [38]. The polyacrylamide chain is highly soluble in water, and its solubility is not influenced by the salt concentration in the aqueous phase. This allows the polyacrylamide block to act as an effective steric stabilizer, whereby, it preferentially interacts with water rather than itself, which prevents particle aggregation in any aqueous based dispersing phase. This also explains why the hydrodynamic size of the 25 nm s-SPIONs is less affected by the dispersing phase.

The TGA and DLS results indicate that a minimal amount of a stabilizing di-block copolymer possessing a suitable anchoring and steric stabilizing block is needed to effectively stabilize our particles in suspension, even at high salt concentrations and in physiological media. The sustainable superparamagnetic properties of SPIONs, especially of the single domain 25 nm core SPIONs, after being coated has been reported in our previous work [38,40].

### 2.2. Biodistribution of *s-SPIONs* in Nude Mice

#### 2.2.1. Macroscopic Observations

**Figure 2:**
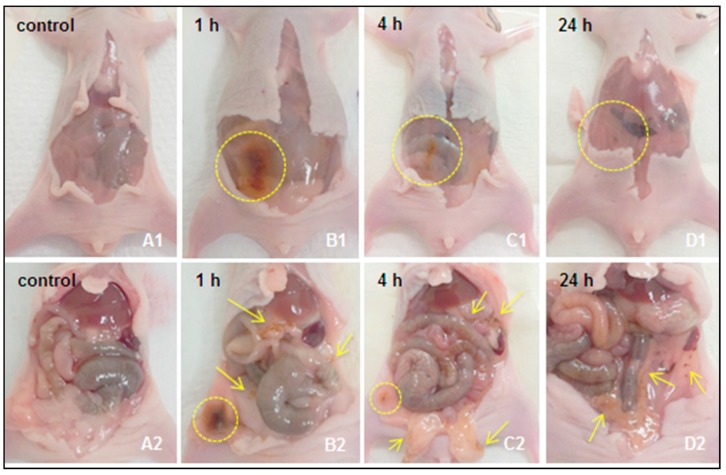
Representative images of the peritoneum following injection of 10 nm s-SPIONs. Photographs were taken at necropsy of the peritoneal cavity before (**A1**–**D1**) and after opening (**A2**–**D2**) the mesentery membrane for the control mouse (**A1**,**A2**) and test mouse at 1 h (**B1**,**B2**), 4 h (**C1**,**C2**), and 24 h (**D1**,**D2**) post-IP injection of the s-SPIONs. The yellow circles and arrows indicate areas of SPION-accumulation visualized by dark brown stained tissues.

Following IP injection of 10 or 25 nm s-SPIONs in PBS (test) or of PBS alone (control), mice were monitored for well-being until euthanasia at time points ranging between 1 h and 7 days post-injection. At the relatively high dose administered (i.e., 90 mg Fe/kg), no adverse effects, signs of illness or weight loss were observed in the mice during this period. Darker colored feces were observed in the test mice that received the s-SPIONs. Peritoneal washings from the test mice were darker in color than those from the control mice up until the 24 h time point. Figure 2 shows representative images of the mice sacrificed at different time points following the IP injection of 10 nm s-SPIONs or PBS alone (Figure 2A). Dark patches in the peritoneal cavity at the site of injection, and in the fatty tissue are clearly evident 1 h after injection, and this is associated with the presence and accumulation of s-SPIONs (Figure 2B, yellow circles and arrows). By 4 h (Figure 2C) and 24 h (Figure 2D) after injection, the accumulation becomes less dense, as indicated by the yellow circles and arrows, clearly showing that the s-SPIONs have dispersed in the peritoneal cavity and moved away from the administration site. There were no s-SPIONs observed at the administration site at the 48 h and 7 day time points. To confirm the clearance of the s-SPIONs from the peritoneal cavity, iron quantification of the peritoneal washings and peritoneal tissues was performed.

#### 2.2.2. Quantitative Measurement of Iron Levels in the Peritoneum Wash, Blood Samples, and Collected Tissue Samples

##### Biodistribution of 10 nm s-SPIONs

**Figure 3:**
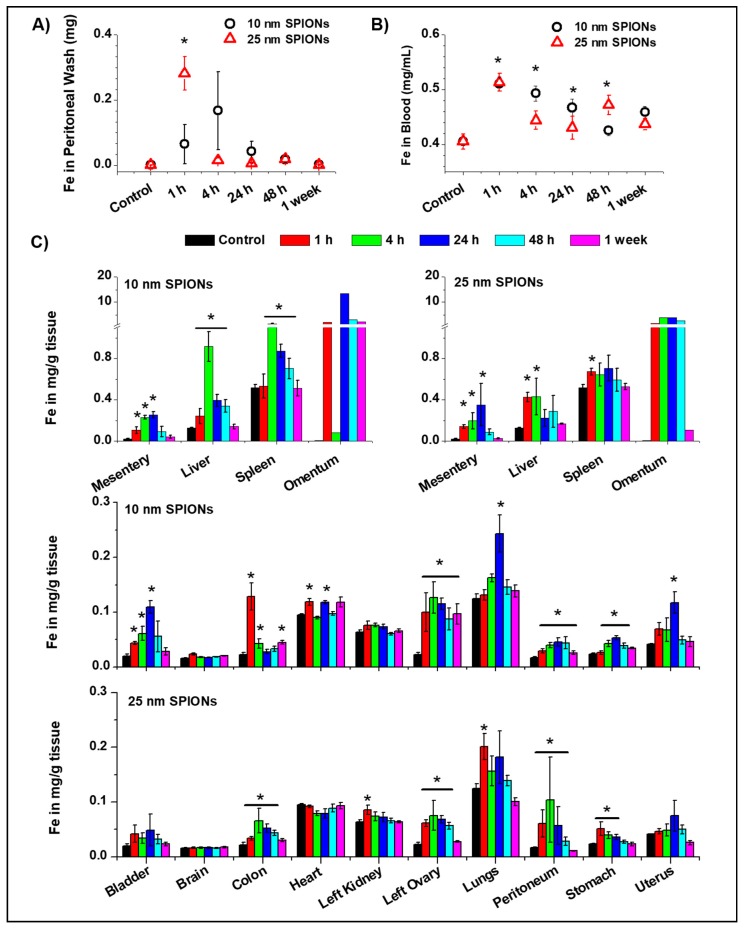
Biodistribution of s-SPIONs determined by AAS iron measurements (**A**) in peritoneal washings; (**B**) in cardiac blood samples, (**C**) in tissues collected at specific time intervals after IP injection of 10 nm and 25 nm s-SPIONs (90 mg of Fe/kg in 200 µL PBS). Data with standard errors from *n* = 4, except for omentum where *n* = 2: * *p* < 0.05 compared to the PBS control.

The biodistribution of s-SPIONs was examined by measuring the iron content in the collected tissues, fluids and blood by AAS. Any increase in the level of iron above control is an indirect indication of the presence of SPIONs in these tissues. Examination of the iron content in the peritoneal washings suggests that the 10 nm s-SPIONs reached a maximal level at 4 h and were cleared by 48 h after administration (Figure 3A). In the cardiac blood samples, iron levels increased by approximately 25% (0.1 mg/mL) above that of control at 1 h after injection before returning back to the control levels by 48 h (Figure 3B).

The 10 nm s-SPIONs were found to be distributed in all tissues examined with no apparent accumulation in the brain or kidneys (Figure 3C). At 1 h post-injection an increase in iron content above control level was detected in the majority of tissues examined. Iron levels in the colon and the heart peaked at 1 h, in the liver, spleen, and ovary at 4 h and in the peritoneal lining, mesentery, omentum, bladder, lungs, stomach, and uterus at 24 h post injection.

Between 48 h and 1 week, the majority of 10 nm s-SPIONs appeared to be cleared from most tissues as the iron levels returned back to control levels. In some tissues such as colon, stomach, and ovaries the level of iron did not completely return back to control levels by one week post-injection. The tissue with the highest concentration 1 week post-injection was the omentum with approximately 1000 µg Fe/g tissue greater than the control. The omentum was also the site with the highest variability with levels between 50 and 10,000 µg Fe/g tissue.

##### Biodistribution of 25 nm s-SPIONs

A similar biodistribution profile was observed for the larger 25 nm core s-SPIONs (Figure 3). Following an increase of 0.3 mg of iron in the peritoneal washing at 1 h post-IP injection, the level dropped back to control level after 4 h suggesting that the 25 nm s-SPIONs were rapidly cleared from the peritoneal cavity. In whole blood, iron levels peaked 1 h after injection with a return towards control levels by 48 h. In both fluids, the 25 nm s-SPIONs appeared to be cleared at a faster rate than the 10 nm s-SPIONs.

Uptake of the 25 nm s-SPIONs was not evident in the brain and heart, and small increases in iron levels were noted in the bladder, kidney and uterus. In other tissues, the iron level peaked at various times—i.e., at 1 h post-injection in the liver, spleen, lung, and stomach, at 4 h in the omentum, ovary, colon, and peritoneum, and at 24 h in the mesentery and uterus. At 1 week post-injection, the iron levels in all tissues examined returned back to control level although in some tissues this was observed as early as 48 h post-injection.

In summary, the 25 nm core s-SPIONs tended to exit the peritoneal cavity faster, and accumulate in the various tissues earlier. Interestingly they were also cleared by the tissues much sooner than the smaller 10 nm s-SPIONs.

#### 2.2.3. Mapping the Distribution of s-SPIONs in Tissues through Iron Staining with Prussian Blue

To examine the intra-organ distribution patterns of iron after treatment with s-SPIONs, Prussian blue staining was performed on tissue sections from both s-SPION-injected and control mice. No positive staining was observed in sections from heart, brain, bladder, stomach, colon, or kidney tissues in the test or control mice. Some iron staining was noted in the adipose tissue surrounding some of these organs, such as in the peri-renal fat of the test mice. Occasional iron staining was noted in the ovarian, lung, and uterine tissue in both the test and control samples.

**Figure 4:**
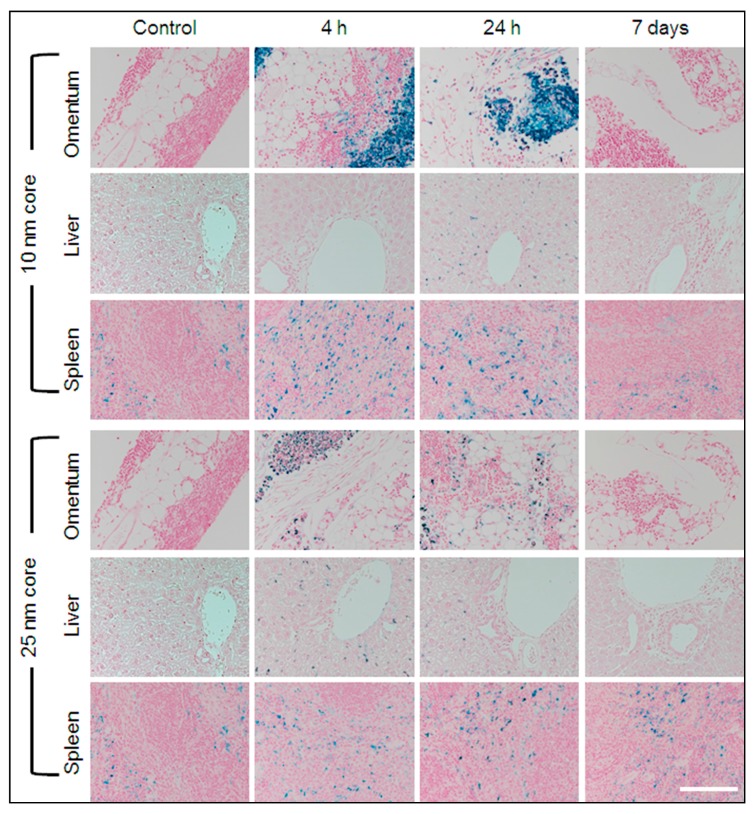
Prussian blue iron staining. Representative sections of tissues identified to have high accumulation of iron are shown for control and s-SPION-injected mice euthanized 4 h, 24 h, or 7 days following injection. High level focal iron staining is evident in omentum 4 and 24 h following injection of 10 nm s-SPIONs. Scale bar = 200 µm.

As expected, positive staining for iron was present in the red pulp of all spleen sections from both the test and control mice (Figure 4). Variable sinusoidal iron staining was observed in kupffer cells of liver sections from s-SPION-injected mice, whereas no staining was present in liver sections from the control mice. For the 10 nm s-SPION-injected mice, Prussian blue staining was evident in liver at 4 h to 24 h and was absent by day 7 (Figure 4). In the 25 nm s-SPIONs-injected mice, positive staining in the liver peaked at 4 h and was absent by 48 h. In the omentum, intense iron staining was observed predominantly but not exclusively in milky spots. The staining in these samples are similar in timing to that observed in the liver—i.e., earlier window of staining than the 10 nm s-SPIONs (Figure 4). These staining results did not correspond with the iron levels at all of the time points (Figure 3C). The differences between the staining and measured iron levels may arise due to the high and focal nature of the s-SPION accumulation, which again indicates the variability in the s-SPION accumulation in the omentum. Overall, the significant finding from this study is that the s-SPIONs are eliminated from the majority of tissues within 7 days after the IP injection.

#### 2.2.4. Examination of Tissues for Signs of Toxicity

##### Histological Assessment

**Figure 5:**
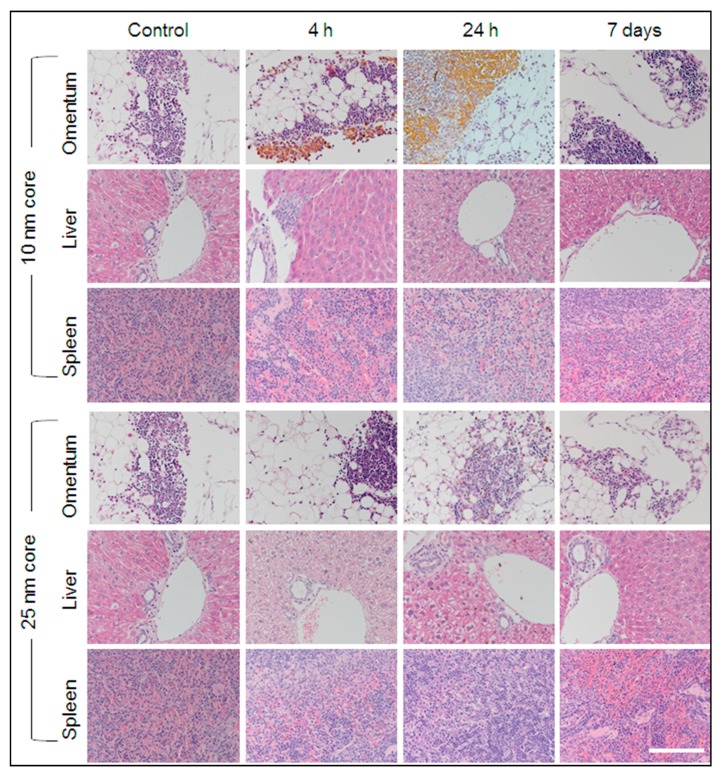
Haematoxylin and Eosin (H&E) staining. Representative sections of tissues identified to have high accumulation of iron are shown for control and s-SPION-injected mice euthanized 4 h, 24 h or 7 days following injection are shown. High s-SPION accumulation is evident by golden brown coloring in omentum 4 and 24 h following injection of 10 nm s-*SPIONs*. Scale bar = 200 µm.

Histological examination of H&E stained tissue sections by a board-certified veterinary pathologist identified signs of inflammation in some tissues from s-SPION-injected mice. As s-SPIONs are foreign to the body, inflammation is considered a normal response. The site of inflammation was most prominent in the omental tissue from s-SPION-injected mice, presenting as an increased macrophage population in the milky spots (Figure 5). The majority of mice from both the control and s-SPION-injected groups showed mild liver pathology consisting of glycogen changes with multifocal moderate karyomegaly, prominent nucleoli, and moderate bi-nucleated cells. These are commonly reported and thought to be associated with caged housing [43]. In addition, rare mild peri-portal inflammation (lymphocytes and neutrophils) was observed in the liver sections from s-SPION-injected mice. This peaked at 4 h in 10 nm s-SPIONs injected mice and 1 h in 25 nm s-SPION-injected mice (see Figure S1) and cleared by 24 h. The histological observation of increased macrophages in some tissues suggests that the s-SPIONs are taken up by macrophages.

##### Immunostaining for Macrophages

**Figure 6:**
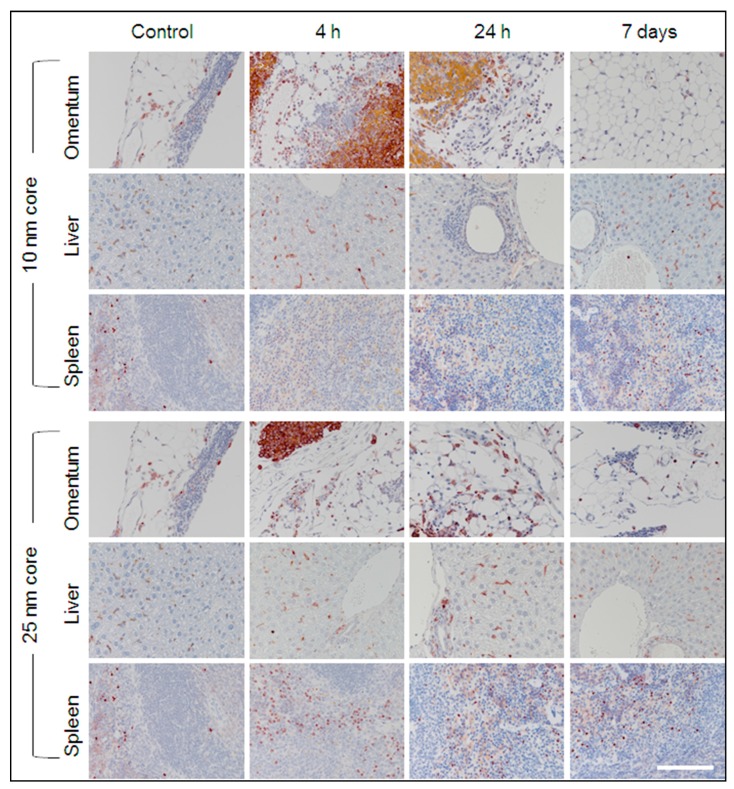
Immunostaining with anti-F4/80 for macrophages. Representative sections of tissues identified to have high accumulation of iron are shown for control and s-SPION-injected mice euthanized 4 h, 24 h, or 7 days following injection. Positive immunostaining is indicated by dark brown color. High focal immunostaining is evident in omentum 4 h following injection of s-SPIONs. High s-SPION accumulation is also evident by golden brown coloring in omentum 24 h following injection of 10 nm s-SPIONs. Scale bar = 200 µm. Isotype control images are shown in Figure S2.

To help determine if s-SPIONs are taken up by macrophages, immunostaining for the macrophage marker F4/80 was performed on sections of liver, spleen, and omentum from s-SPION-injected and control mice (Figure 6 and Figure S2). Occasional positive staining was detected in control tissues. This pattern was also evident in liver and spleen sections from s-SPION-injected mice, although there was no positive immunostaining in the periportal regions. An increase in highly focal positive immunostaining was observed in omental tissues from s-SPION-injected mice. However, it was critical to distinguish positive dark brown immunostaining from the golden brown, s-SPION accumulation (Figure S2). In all examined tissues, the increased macrophage levels were transient and returned to control levels within 7 days. These results support a role for macrophages in uptake and clearance of s-SPIONs.

#### 2.2.5. Clearance Pathway of s-SPIONs

##### Excretion of s-SPIONs via Feces

Spot fecal samples were collected from all mice at the time of sacrifice to measure the iron content. Iron levels peaked at 1 to 4 h for mice injected with 10 nm s-SPIONs and 1 h for mice injected with 25 nm s-SPIONs (Table 1). After this time, iron levels returned to basal control levels. As only spot samples were tested, it was not possible to determine the total percentage of s-SPIONs excreted via feces. However, these results show that clearance of s-SPIONs from the body following IP administration involves rapid fecal excretion.

##### Excretion of s-SPIONs via Macrophages

The above results from the iron uptake and Prussian blue staining demonstrate that the s-SPIONs accumulate transiently in the liver and spleen and suggest that the s-SPIONs are readily engulfed and redistributed by macrophages. To further characterize and confirm the rapid uptake of s-SPIONs by macrophages, in vitro experiments were performed using the murine RAW 264.7 macrophage cell line. Macrophages were sampled at time intervals after s-SPION treatment to quantify the iron concentration and to visualize the iron uptake by light microscopy or TEM.

**Figure 7:**
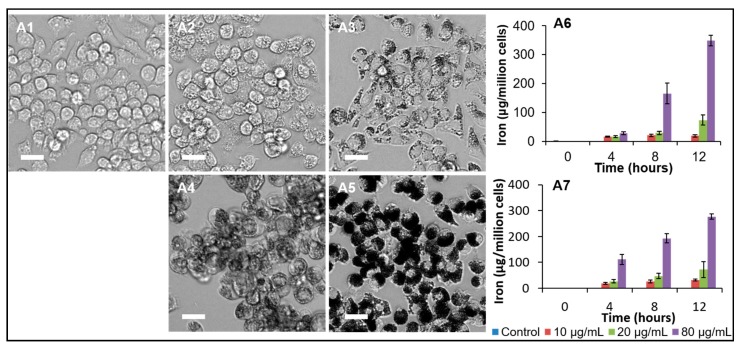

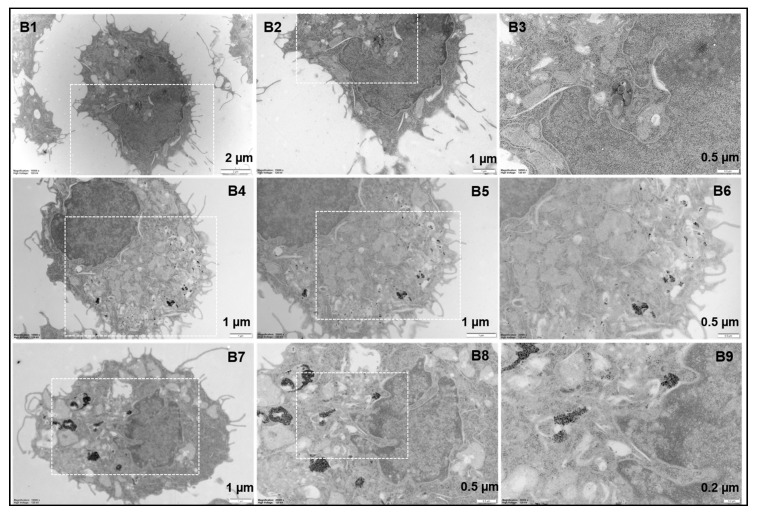
Characterization of s-SPION uptake in RAW264.7 macrophages. (**A1**–**A5**) bright-field microscope images of cells grown in monolayers untreated (**A1**) and treated with 10 nm (**A2**,**A3**) or 25 nm (**A4**,**A5**) s-SPIONs for 24 h at 25 µg/mL (**A2**,**A4**) and 250 µg/mL (**A3**,**A5**). Scale bar: 40 µm. (**A6**,**A7**) iron levels in the cells grown in suspension when treated with 10 nm (**A6**, *n* = 3/time point) or 25 nm (**A7**, *n* = 3/time point) s-SPIONs. (**B**) TEM images of control macrophages (**B1**–**B3**) and macrophages treated with 10 nm (**B4**–**B6**) and 25 nm (**B7**–**B9**) s-SPIONs at 20 µg/mL for 24 h.

As shown in Figure 7A, macrophages are capable of sequestering large amounts of s-SPIONs, which appeared as dark spots inside cells grown in a monolayer (10 nm s-SPIONS Figure 7(A2,A3); 25 nm s-SPIONS Figure 7(A4,A5)). At the same iron dose the cells treated with the 25 nm s-SPIONs look darker than those treated with 10 nm s-SPIONs due to the size of the cores; the 25 nm cores being more electron dense will reflect more light than smaller cores. However, there was no significant difference in iron accumulation when cells grown in suspension were treated with the different sized s-SPIONS (Figure 7(A6,A7)) and the cells were able to accumulate iron at concentrations as high as 300 µg/10^6^ cells in 12 h. This is approximately 1000 times higher than non-phagocytic cells tested in the same manner [35].

#### 2.2.6. Biodistribution of s-SPIONs in Tumor-Bearing Mice

**Figure 8:**
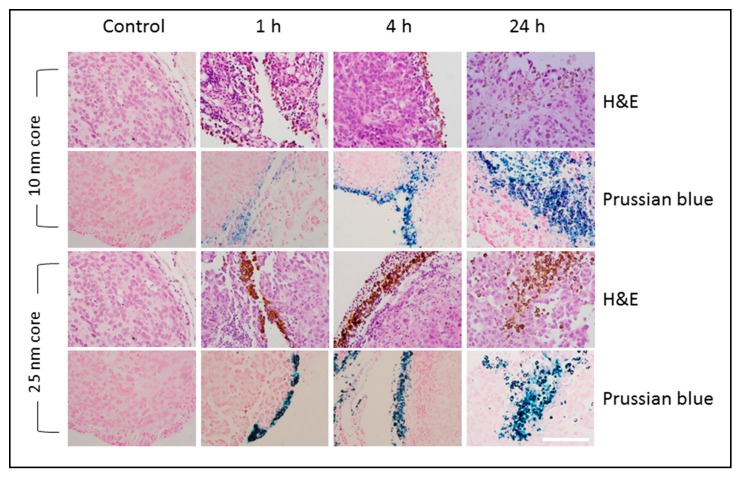
Distribution of s-SPIONs to ovarian tumors. Representative sections of tumor tissues are shown for control and s-SPION injected mice euthanized at 1, 4, or 24 h following injection. High s-SPION accumulation is evident by the golden brown coloring in the H&E-stained sections on the surface of tumors at 1 and 4 h following injection, and infiltrating into the tumor at 24 h following injection. Prussian blue iron staining is also evident. Scale bar = 200 μm.

We investigated the biodistribution of our s-SPIONs in mice with tumors generated by IP injection of SKOV3 human ovarian cancer cells. At necropsy, mice were found to have tumors throughout the peritoneal cavity, including the omentum. As shown in Figure 8, s-SPIONs were detected on the surface of tumors from 1 h post-injection. Consistent with the biodistribution results in healthy mice, 25 nm s-SPIONs accumulated at higher levels on the tumor surface at earlier time points than the 10 nm s-SPIONs. At 24 h both 10 and 25 nm s-SPIONs were still visible on the surface of tumors, with regions demonstrating infiltration into the tumor. These observations were supported by Prussian blue iron staining.

## 3. Discussion

The stability of nanoparticles governs their biodistribution and determines their toxicity in vitro and in vivo. We previously reported that our s-SPIONs are highly stable and well dispersed in fresh human red blood cells [44]. The prolonged stability of s-SPIONs in vitro under physiological conditions is favorable for potential biomedical applications. In the current study, we undertook a systematic examination of the in vivo biodistribution and clearance profile of the 10 and 25 nm s-SPIONs following IP injection to healthy nude mice. These s-SPIONs enter the circulatory system with a therapeutic window of 1–4 h. While we administered a high, but clinically relevant dose of s-SPIONs our results indicate that the particles did not aggregate or remain in the abdominal cavity. This is evident macroscopically, microscopically, and quantitatively (by iron levels). By 24 h post injection, s-SPIONs were no longer evident macroscopically at the injection point. The decrease in iron content in both peritoneal washings and in the peritoneum tissues further supports the clearance of the s-SPIONs from the peritoneal cavity. While the macroscopic images cannot differentiate between the accumulation and aggregation of the s-SPIONs, the fact that the s-SPIONs cleared from the injection site strongly suggests that the formation of aggregates is unlikely; aggregate formation would preclude the clearance of the s-SPIONs, becoming lodged within the tissues due to their large size.

With the exception of the brain and kidneys, the s-SPIONs dispersed to the major organs, and were cleared within one week as demonstrated by the return to basal or near basal iron levels in the major organs. This clearance, with the return to basal iron levels, further indicates that the s-SPIONs remained stable rather than forming aggregates. The formation of large aggregates would prevent their effective clearance from the body. The s-SPIONs do not appear to penetrate the intact blood brain barrier of the healthy nude mice used in this study. However, further studies are required to determine the s-SPION dispersal in situations where the blood brain barrier is transiently damaged.

A major concern for nanoparticles larger than 8 nm, the accepted threshold for filtration by the glomerulus, is the development of renal fibrosis due to the accumulation and retention of the nanoparticles in the kidneys [45]. There was no evidence of the 10 nm s-SPIONs within the kidneys. However, bladder iron levels increased, suggesting some penetration of the s-SPIONs into the bladder independent of filtration through the kidney. For the 25 nm s-SPIONs, a small transient increase in iron levels after 1 h was detected for the kidney, which may be due to some accumulation in the peri-renal fat. While we cannot exclude that the s-SPIONs may have entered the kidneys and the bulk cleared before the first time measured point measured (1 h), this is unlikely as the s-SPION size is above the accepted threshold for glomerular filtration. With negligible detection of s-SPIONs in the kidney, the potential for renal fibrosis is reduced. However, a full toxicological assessment is required to confirm this. At the high dose of s-SPIONs (90 mg Fe/kg body weight) administered to the healthy nude mice in this study, which translates to a suitable dose in human, there is no evidence of any tissue damage. Histological observation and in vitro studies suggest rapid clearance of s-SPIONs by macrophages.

The cellular microenvironment, including the presence of iron has a direct influence on macrophage polarization. High intracellular iron levels generally lead to the activation of pro-inflammatory (M1) macrophages that sequester iron as part of their bacteriostatic mechanism. This is accompanied by a reduction in anti-inflammatory (M2) macrophages, which have a high iron releasing capacity [46]. Long-term, this imbalance in M1/M2 macrophages can lead to tissue breakdown and inflammatory diseases. However, M1 macrophages also have anti-tumor properties that may be able to be transiently harnessed either directly or indirectly [47]. We have previously reported that our s-SPIONs enhance the uptake and efficacy of chemotherapeutics in vitro [35]. Inducing an M1 anti-tumor phenotype during delivery of anti-cancer agents in vivo may further boost treatment efficacy. At the same time, preventing the switch from M1 to M2 macrophages may have an added benefit by inhibiting M2 tumor-promoting activities.

The tumor-homing properties of macrophages coupled with their capacity to carry nanoparticles can also be exploited for targeting anti-cancer therapy. We observed an exceptionally high, but variable, accumulation of s-SPIONs and macrophages in the omentum. This tissue is the most common metastatic site for ovarian cancer [48], suggesting even greater accumulation of nanoparticles in cancerous omentum and a possible mechanism to target anti-cancer therapies to this tissue. Therefore, we determined whether our s-SPIONs would localize to tumors in a metastatic model of ovarian cancer. Both 10 nm and 25 nm s-SPIONs were seen to be present on the surface of tumors from 1 h post injection, with infiltration into tumors seen at 24 h. This supports our previous in vitro studies that suggest co-administration of our s-SPIONs may enhance the delivery of chemotherapy to tumors and warrants further studies.

## 4. Materials and Methods

### 4.1. Materials

RAFT agents, 2-[(butylsulfanyl)carbonothioyl]sulfanyl propanoic acid (C_4_-RAFT) and methoxy-polyethylene glycol modified 2-[(butylsulfanyl)carbonothioyl] sulfanyl propanoic acid (RAFT-PEO), were kindly provided by Algi Serelis (DuluxGroup; Clayton, VIC, Australia). Iron(II) chloride tetrahydrate (99%), Ion(III) chloride hexahydrate (98%), acrylamide (AAm, ≥98%), *N*-(3-dimethylaminopropyl)-*N’*-ethylcarbodiimide hydrochloride (EDC HCl, ≥98%), *N*-hydroxysuccinimide (NHS, ≥99%), *N*-Boc-ethylenediamine (≥98%), 4(dimethylamino)pyridine (DMAP, ≥99%) and hydrochloric acid (HCl, 30–35%, TraceSELECT^®^ Ultra) were purchased from Sigma-Aldrich. Iron(III) nitrate nonahydrate (99%), anhydrous magnesium sulfate (MgSO_4_, ≥98%), nitric acid (HNO_3_, 65%, Suprapur^®^, Millipore) and silica gel were obtained from Merck (Darmstadt, Germany). Ammonium hydroxide (28% NH_3_ in water, *w*/*w*), sodium hydroxide (NaOH) pellets (≥98%), dichloromethane (DCM, ≥98%), hexane (≥98%), and ethyl acetate (≥98%) were purchased from Ajax Finechem (Sydney, Australia). 1,4-Dioxane (Fluka, Sigma-Aldrich), monoacryloxyethyl phosphate (MAEP, ≥98%, PolySciences Inc., Warrington, PA, USA), 4,4′-azobis(4-cyanovaleric acid) (V-501, Wako Pure Chemicals C. Ltd., Tokyo, Japan), deuterium oxide (D_2_O, 99.90%, Cambridge Isotope Laboratories, Novachem Pty. Ltd., Heidelberg West, VIC, Australia), and deuterated chloroform (CDCl_3_, 99.8%, Cambridge Isotope Laboratories) were used as received.

### 4.2. Characterization Methods

#### 4.2.1. NMR Spectroscopy

A 500 MHz Bruker Ultra Shield™ (Bruker Pty. Ltd. Alexandria, NSW, Australia) spectrometer was used to record ^1^H-NMR, and ^13^C-NMR spectra of RAFT-NH_2_ with CDCl_3_ as the solvent. The polymerizations of AAm and MAEP were determined by ^1^H-NMR spectroscopy in D_2_O using a 300 MHz Bruker Ultra Shield spectrometer. The monomer conversion was determined by the integration of the trioxane peak at 5.1 ppm and the vinylic monomer proton peaks at 6.6, 6.3, and 5.7 ppm.

#### 4.2.2. Dynamic Light Scattering (DLS) and Transmission Electron Microscopy (TEM)

A DLS instrument (Zetasizer nano series, helium-neon laser at 633 nm, 40 mW, Malvern Instruments Ltd., Malvern, UK) with a detection angle of 173°, was used to measure the particle size at 25 °C. The particles were dispersed in Milli-Q (Merck Millipore, Bayswater, VIC, Australia) water at a concentration of 0.5 mg/mL prior to measurement. The stability of s-SPIONs under physiological conditions was studied by using a dispersion of the particles in PBS.

The particle size and size distribution of s-SPIONs were also investigated using TEM (JEM-1400 JEOL (Australasia) Pty. Ltd., Frenchs Forest, NSW, Australia), accelerating voltage 120 kV). A diluted sample (approximately 10 µg/mL in water) of s-SPIONs was drop-cast onto a 200 mesh carbon-coated Formvar™ copper TEM grid and air-dried before imaging.

#### 4.2.3. Thermogravimetric Analysis (TGA)

Thermogravimetric analysis (TGA, TA Instrument, Waters Australia Pty. Ltd., Rydalmere, NSW, Australia) was used to quantify the amount of polymeric stabilizer incorporated onto the s-SPIONs. A minimum amount of 10 mg dry weight of each sample was loaded onto the instrument. The sample was equilibrated at 100 °C for 10 min, followed by a gradual temperature ramp to 700 °C at 10 °C/min.

#### 4.2.4. Atomic Absorption Spectroscopy (AAS)

The iron content in the s-SPIONs was measured using an AAS instrument (Varian AA800 spectrometer, with acetylene/air flame atomization). An iron standard solution (1000 mg/L in HNO_3_, Merck) was used to prepare 2, 4, 6, 8, and 100 ppm standard solutions for the calibration curve. Samples of dried s-SPIONs were digested in Suprapur^®^ HNO_3_ (500 µL for each sample, Merck-Millipore) at 70 °C, followed by dilution with 0.1 M HCl (Merck) to a final volume of 5.0 mL prior to measurement.

Either graphite furnace atomic absorption spectroscopy (GF-AAS, Agilent 240FS AA spectrometer with a Zeeman graphite tube atomizer; Agilent Technologies Australia, Mulgrave, VIC, Australia) or flame-AAS (Varian AA800 spectrometer, with acetylene/air flame atomization; Agilent Technologies Australia) were used to quantify iron content in the collected frozen tissues. For GF-AAS, a standard solution of iron in 1% (*v*/*v*) HNO_3_ at a concentration of 25 ppb was prepared from a commercial iron standard (Merck Millipore, 1000 mg/L in HNO_3_,) for a calibration curve at 5, 10, 15, and 20 ppb.

### 4.3. Synthesis of Sterically Stabilized SPIONs

#### 4.3.1. The Raw Magnetic Ferrofluids

Two different core sizes of maghemite iron oxide (γ-Fe_2_O_3_) with average diameter of 10 and 25 nm were used in this study. The 25 nm cores were kindly donated from Sirtex Technologies Pty. Ltd. (North Sydney, NSW, Australia). The 10 nm cores were synthesized in house using Massart’s co-precipitation method, [49] and was reported in our previous publications [35,38].

#### 4.3.2. Synthesis of *N*-Boc-ethylenediamine C_4_-RAFT Agent (tert-butyl (2-(2-(((butylthio)carbonothioyl)thiopropanamido)ethyl)carbamate)

C_4_-RAFT (7.00 g, 29.4 mmol), EDC .HCl (6.73 g, 35.1 mmol), and DMAP (0.430 g, 3.5 mmol) were dissolved in DCM 350 mL in a round bottom flask. The dark red solution was cooled down to 0–5 °C using an ice bath. A solution of *N*-Boc-ethylenediamine (5.19 g, 32.4 mmol) in DCM (10 mL) was then added to the mixture over a period of approx. 5 min. After stirring for about 30 min at 0 °C, the reaction was left to warm to room temperature where it was stirred for another hour. The solution, which turned yellow during the process, was then washed with water (3 × 100 mL) in a separating funnel. The organic phase was collected, dried over MgSO_4_ and filtrated. The DCM was removed by rotary evaporation. The crude product was then purified by silica gel column chromatography using hexane/ethyl acetate (1:1 *v/v*) as the. The middle fraction presenting with an intense yellow color was collected and evaporated to dryness. The obtained solid was then purified by recrystallization from hexane, washed with cold hexane, and finally dried in a vacuum oven (40 °C, 24 h) to yield the pure product (7.18 g, 18.9 mmol, Yield: 60%). ^1^H-NMR and ^13^C-NMR (Figure S3) were used to confirm the chemical structure of the product. The purity was also ascertained by mass spectrometry. HRMS-ESI (*m/z*): [M + Na]^+^ calculated for C_15_H_28_N_2_O_3_S_3_, 403.11543; found, 403.11542.

#### 4.3.3. Synthesis of NH_2_ End Functionalized Macro-RAFT Diblocks: Poly(monoacryloxyethyl phosphate)_10_-block-poly(acrylamide)_20_ (RAFT-MAEP_10_-AAm_20_-NH_2_) and poly(monoacryloxyethyl phosphate)_10_-block-poly(acrylamide)_60_ (RAFT-MAEP_10_-AAm_60_-NH_2_) Using the *N*-Boc-Ethylenediamine C_4_-RAFT Agent

The diblocks were synthesized in a water and dioxane solvent mixture, following standard procedures published previously [35,38], and further outlined in the Supporting Information. During the formation of the MAEP block, the protecting *N*-Boc-ethylenediamine group was deprotected, resulting in the NH_2_ functionalized end group. This was supported by the replacement of the 1.3 ppm peak by the 1.2 ppm peak in the ^1^H-NMR spectra). The diblock copolymers were precipitated in cold methanol, collected by filtration and dried in a vacuum oven at 40 °C. ^1^H-NMR and ^31^P-NMR were used to quantify the number of monomer units in each block, and are shown in Figures S4 and S5 respectively.

#### 4.3.4. Synthesis of MPEG End Functionalized Macro-RAFT Diblocks: Poly(ethylene oxide)_17_-block-poly(monoacryloxyethyl phosphate)_10_ Macro-RAFT Agent (RAFT-MAEP_10_-MPEG) and Poly(ethylene oxide)_17_-block-poly(acrylamide)_40_-block-poly(monoacryloxyethyl phosphate)_10_ Macro-RAFT Agent (RAFT-MAEP_10_-AAm_40_-MPEG)

The MPEG-functionalized RAFT agent (RAFT-MPEG) agent was kindly donated by the DuluxGroup, Australia. The diblocks were synthesized in a solvent mixture of water and dioxane following standard procedures published previously [35,38], and are outlined in the Supporting Information. The macro-RAFT solution obtained was concentrated by rotary evaporation and dialyzed against MQ-water using 2000 molecular weight (MW) cut-off dialysis tubing. ^1^H-NMR and ^31^P-NMR were used to quantify the number of monomer units in each block, and are shown in Figures S6 and S7 respectively.

#### 4.3.5. Stabilization of the γ-Fe_2_O_3_ with the Steric Stabilizers

For a dispersion of 10 nm SPIONs (200 mL, 1 wt. %, pH = 4.0), an aqueous mixture containing 1 wt. % of *RAFT-MAEP*_10_-*MPEG* (1.82 g dry weight) and *RAFT-MAEP*_10_-*AAm*_20_-*NH*_2_ (0.108 g dry weight), pH adjusted to 4.0, was used for coating. To coat the 25 nm SPIONs (190 mL, 1 wt. %, pH = 4.0), a mixture of *RAFT-MAEP*_10_-*AAm*_20_-*MPEG* (3.89 g dry weight) and *RAFT-MAEP*_10_-*AAm*_40_-*NH*_2_ (0.068 g dry weight), pH adjusted to 4.0, was used. The s-SPIONs were separated from free polymers by ultracentrifugation (30,000 rpm, 40 min, 3 times) and then dispersed in water for further characterization.

The s-SPIONs were dispersed in a PBS, filtered through a 0.22 µm sterile syringe filter, and maintained under sterile conditions prior to the in vitro and in vivo studies. The final concentration was 7 mg iron/mL PBS for both types of s-SPIONs.

### 4.4. In Vivo Experiments

#### 4.4.1. Ethics Statement

All animal experiments were approved by the Northern Sydney Local Health District Animal Ethics Committee (Protocol 1310_003A, approved 31 October 2013) and were conducted in accordance with the Australian National Health and Medical Research Council (NHMRC) guidelines and conformed to the Australian Code for the Care and Use of Animals for Scientific Purposes.

#### 4.4.2. Animals

Six to eleven week old BALB/c-*Foxn1^nu^*/Arc (nude) mice, with an average weight of 17 g each, were obtained from the Animal Resources Centre, Perth, WA, Australia and housed in the Kearns Small Animal Facility, Kolling Institute in micro-isolater caging with 12 h light/dark cycles and sterile food and water *ad libitum*. Mice were acclimatized for at least one week prior to experiments.

#### 4.4.3. Biodistribution in Healthy Mice

Mice underwent IP injections of s-SPIONs in 200 µL PBS, equivalent to a total amount of 1.4 mg Fe per mouse or an average of 90 mg Fe/kg body weight. Control mice were injected with 200 µL PBS alone. Groups of 4 mice were sacrificed at 1 h, 4 h, 24 h, 48 h, and 7 days post-injection. Mice were monitored daily for well-being. At euthanasia (asphyxiation) mice underwent lavage with 5 mL PBS IP injection and the peritoneal washings were collected and snap frozen in liquid nitrogen. Photos were taken of the test mice at the 1, 4, and 24 h time points with the peritoneal cavity open. The following tissues were collected and divided into 10% neutral buffered formalin for histology and snap frozen in liquid nitrogen for iron quantification: liver, spleen, kidneys, colon, bladder, peritoneum, ovaries, uterus, stomach, heart, lungs, brain, and mesentery. The omentum was too small to be divided, and therefore the tissue collected from each mouse was used either for histology or iron quantification. Cardiac blood and spot feces were also collected and snap frozen.

#### 4.4.4. Biodistribution in Tumor-Bearing Animals

Prior to injection into nude mice, SKOV3 ovarian cancer cells stably expressing luciferase were grown in RPMI + 10% fetal bovine serum and 20 mM l-glutamine and maintained in a humidified incubator at 37 °C and 5% CO_2_. Mice were injected IP with 1 × 10^7^ cells and underwent bioluminescent imaging once a week to monitor for tumor growth. For imaging, mice were injected IP with 300 mg/kg d-Luciferin (Perkin-Elmer, Boston, MA, USA) fifteen minutes prior to imaging on an in vivo FX Pro (Bruker). Mice that demonstrated visible tumors after imaging were then used to investigate SPION distribution. As previously, mice were injected IP with s-SPIONs in 200 µL PBS, equivalent to a total amount of 1.4 mg Fe per mouse or an average of 90 mg Fe/kg body weight. Control mice were injected with 200 µL PBS alone. Groups of 3 mice were sacrificed at 1 h, 4 h, and 24 h post-injection. Tumors were collected into 10% neutral buffered formalin for histology.

#### 4.4.5. Preparation of Samples for Iron Quantification

All sample tubes were washed with 0.1% (*v*/*v*) HNO_3_ before use. All collected tissues and blood samples were weighed, except the peritoneal wash that was evaporated, then digested with 1.0 mL of Ultrapur 69% HNO_3_ at 70 °C, for >1 h and diluted to a final volume of 5.0 mL with a 0.1% (*v*/*v*) low trace metal HNO_3_ solution. The iron content of the prepared solutions was measured by flame AAS for samples with a high Fe content, or by a GF-AAS for samples with an Fe concentration below 2 ppm.

#### 4.4.6. Histology and Immunohistochemistry

Collected tissues were fixed in formalin overnight, transferred to 70% ethanol, processed and paraffin embedded. 4 μm sections were cut and mounted for H&E staining using standard techniques. Prussian blue staining was performed to detect ferric iron ions in the tissue sections. Briefly, sections were deparaffinized and rehydrated, then incubated with a 1:1 mixture of 2% potassium ferrocyanide and 2% hydrochloric acid for 10 min. Slides were rinsed in distilled water and the nuclei counterstained with nuclear fast red (Point of Care Diagnostics Scientific, Artarmon, NSW, Australia). Immunohistochemistry was performed to detect the macrophage marker F4/80. Sections were deparaffinized and rehydrated before antigen unmasking using a target retrieval solution (S1699, pH 6.0; Dako, Carpenteria, CA, USA) in a boiling water bath for 20 min. Endogenous peroxidase activity was quenched with a 0.3% hydrogen peroxide solution. Sections were incubated for 1 h at room temperature with 1:50 anti-F4/80 (BM8) monoclonal rat antibody (Santa Cruz Biotechnologies, Dallas, TX, USA) or the isotype control (Rat IgG2a Isotype control Cat # 559073, BD Pharminogen, Franklin Lakes, NJ, USA). A labelled polymer horseradish peroxidase anti-rat detection system was used according to the manufacturer’s instructions (Rat-on-Mouse HRP-Polymer; Biocare Medical, Concord, CA, USA). ImmPACT NovaRED peroxidase was used as substrate (Vector Laboratories, Burlingame, CA, USA). Slides were counterstained with Mayer’s Haematoxylin.

### 4.5. In Vitro Studies

#### 4.5.1. Cell Culture

The murine macrophage cell line RAW264.7 (European Collection of Authenticated Cell Cultures (ECACC), Catalogue No. 91062702; Sigma-Aldrich) was used to determine the cytotoxicity and uptake of s-SPIONs in vitro. Cells were maintained as confluent suspensions in DMEM (Gibco, 10566-016; Thermo Fisher Scientific, North Ryde, NSW, Australia), supplemented with 10% (*v*/*v*) Fetal Calf Serum. The cell suspension was incubated under standard culturing conditions of 37 °C with 5% (*v*/*v*) CO_2_ under humidified conditions and gently mixed on top of an Orbi-Shaker (Benchmark Scientific, Sayreville, NJ, USA, 19 mm orbit motion, 80 rpm). The cell suspension was collected in a centrifuge tube and spun down at approximately 1000 g for 3 min. The supernatant was discarded and the cells were resuspended in fresh growth medium. Cells were counted on a haemocytometer with 0.2% (*v*/*v*) trypan blue (Gibco, 15250-061) and an appropriate number of cells were seeded for further experiments and sub-culturing.

#### 4.5.2. Preparation of Cell Samples for Iron Quantification

RAW264.7 cells were seeded at a cell density of 3 × 10^5^ cells/mL into each well of a 6-well plate and incubated for 24 h under standard culturing conditions. The cells were then dosed with 10, 20 or 80 ppm s-SPIONs for 24 h, washed 3 times with 2 mL PBS, detached from the flask by 0.25% (*w*/*v*) trypsin and collected into a 1.8 mL centrifuge tube. The cell suspension was centrifuged (2000× *g)* for 3 min, at room temperature), the cell pellets resuspended in 1 mL of PBS before being recentrifuged. This step was repeated 3 times. After removal of the supernatant following the last centrifugation, the tubes containing the cell pellets were dried in a vacuum concentrator (Eppendorf South Pacific, Pty. Ltd., Macquarie Park, NSW, Australia; at 60 °C, 1 h, 250 g centrifugation). The samples were digested with 200 µL of concentrated nitric acid (Suprapur^®^, 65%), and then diluted to a final volume of 1 mL with a 1% (*v*/*v*) nitric acid for analysis using AAS.

RAW264.7 cells were also grown in suspension and cultures with a density of 5 × 10^5^ cells/mL were dosed with 10, 20, or 80 ppm s-SPIONs for 24 h. Following this, 1 mL aliquots of the cell suspension were collected and centrifuged (2000× *g*) for 3 min, at room temperature. The cell pellets were washed with 1 mL of PBS six times, and then prepared in similar manner as the adherent monolayer cells for analysis on the AAS.

#### 4.5.3. Preparation of Cell Samples for TEM

RAW264.7 cells were seeded at a cell density of 3 × 10^5^ cells/mL into each well of a 6-well plate containing Thermanox™ coverlip (Proscitech, Kirwan, QLD, Australia) and incubated for 24 h under standard culturing conditions. The cells were then dosed with 20 ppm s-SPIONs for 24 h, washed 3 times with PBS and prepared for TEM following the standard procedure as described previously [35,38]. After embedding in epon resin, thin sections (70–90 nm) were cut using an ultramicrotome and mounted on a TEM grid, followed by post-staining with heavy metals. TEM was conducted on JEM-1400 at 120 kV.

### 4.6. Statistical Analysis

Data was expressed as a mean ± standard error of the mean (SEM) with a sample size (*n*). Significance was determined from the average of two groups using the independent samples *t*-test and for groups of three or more by two-way analysis of variance (ANOVA). When two-way ANOVA was utilized, the post-hoc analysis for pairwise comparison was the Bonferroni test. An α value of 5% (*p* < 0.05) was considered statistically significant, and is indicated in the figures as * (*p* < 0.05) where appropriate.

## 5. Conclusions

To the best of our knowledge, this is the first report of the in vivo biodistribution of physiologically stable aqueous-based superparamagnetic iron oxide nanoparticles from single domain cores of 25 nm in diameter. The broad biodistribution, high stability and rapid clearance of these s-SPIONs confirm their potential for bio-medical applications. Furthermore, we demonstrate their ability to localize to tumors. Future directions include in vivo assessment of the s-SPIONs as adjuncts to improve drug uptake.

## Figures and Tables

**Table 1:** Iron content in feces samples collected from control and s-SPION injected mice. Iron quantified by AAS.

Time Point	Total Iron in Feces (µg) from Mice Injected with	
	10 nm s-SPIONs	25 nm s-SPIONs
Control	10.8 ± 2.5	10.8 ± 2.5
1 h	28.4 ± 17.7	20.6 ± 13.9
4 h	28.0 ± 14.0	11.0 ± 3.7
24 h	7.2 ± 2.4	11.8 ± 1.5
48 h	9.5 ± 1.1	13.4 ± 2.8
1 week	11.5 ± 0.3	8.6 ± 2.1

## Supplementary Materials

Supplementary materials can be found at www.mdpi.com/1422-0067/19/1/205/s1.

## Abbreviations

SPION	superparamagnetic iron oxide nanoparticles
s-SPIONs	sterically stabilized superparamagnetic iron oxide nanoparticles
IONP	iron oxide nanoparticle
IP	intraperitoneal injection
IV	intravenous injection
AAS	atomic absorption spectroscopy
GF-AAS	graphite furnace—atomic absorption spectroscopy
RAFT	reversible addition fragmentation chain transfer
MPEG	methoxypolyethylene glycol
AAm	acrylamide
MAEP	monoacryloxyethyl phosphate
NMR	nuclear magnetic resonance
TEM	transmission electron microscopy
TGA	thermogravimetric analysis
DLS	dynamic light scattering
ROS	reactive oxygen species
PBS	phosphate-buffered saline
H&E	haematoxylin and eosin
