# Supplementary material for: Biodistribution and Clearance of Stable Superparamagnetic Maghemite Iron Oxide Nanoparticles in Mice Following Intraperitoneal Administration
Source: Int J Mol Sci. 2018 Jan 10;19(1):205. doi: 10.3390/ijms19010205 (PMC5796154; doi:10.3390/ijms19010205)
Supplement: Supplementary file 1 [file ijms-19-00205-s001.pdf]

International Journal of
Molecular Sciences

Biodistribution and Clearance of Stable Superparamagnetic Maghemite Iron Oxide Nanoparticles in Mice Following Intraperitoneal Administration

Binh T. T. Pham, Emily K. Colvin, Nguyen T. H. Pham, Byung J. Kim, Emily S. Fuller, Elizabeth A. Moon, Raphael Barbey, Samuel Yuen, Barry H. Rickman, Nicole S. Bryce, Stephanie Bickley, Emily K. Colvin, Stephen K. Jones, Viive M. Howell and Brian S. Hawkett

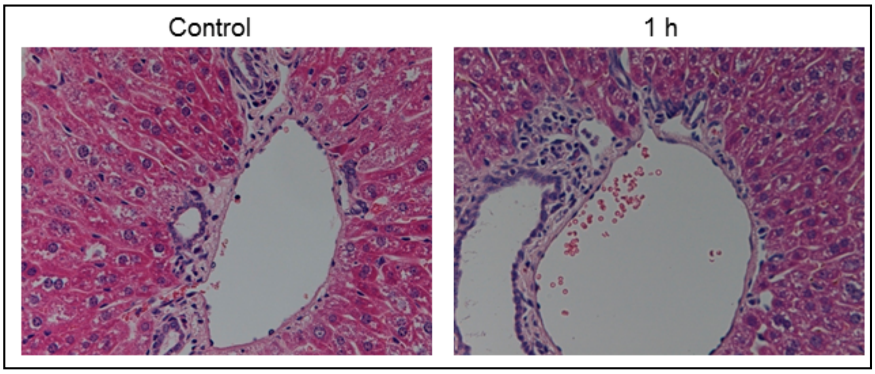

Figure S1. H&E stained sections of livers showing rare mild peri-portal inflammation. Representative sections are shown from a control mouse and a mouse euthanized 1 h after treatment with 25 nm s-SPIONs; original magnification: 400×.

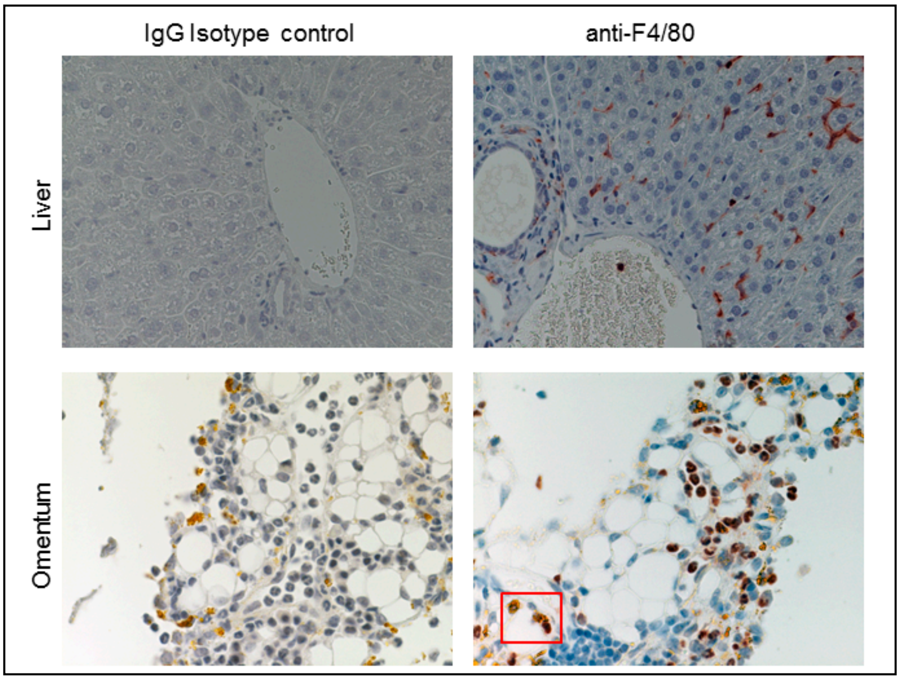

Figure S2. Isotype control and anti-F4/80 immunostaining in liver and omentum. The red inset shows the different intensities of iron (light, golden) and F4/80 (dark); original magnification: 400× (liver), 600× (omentum).

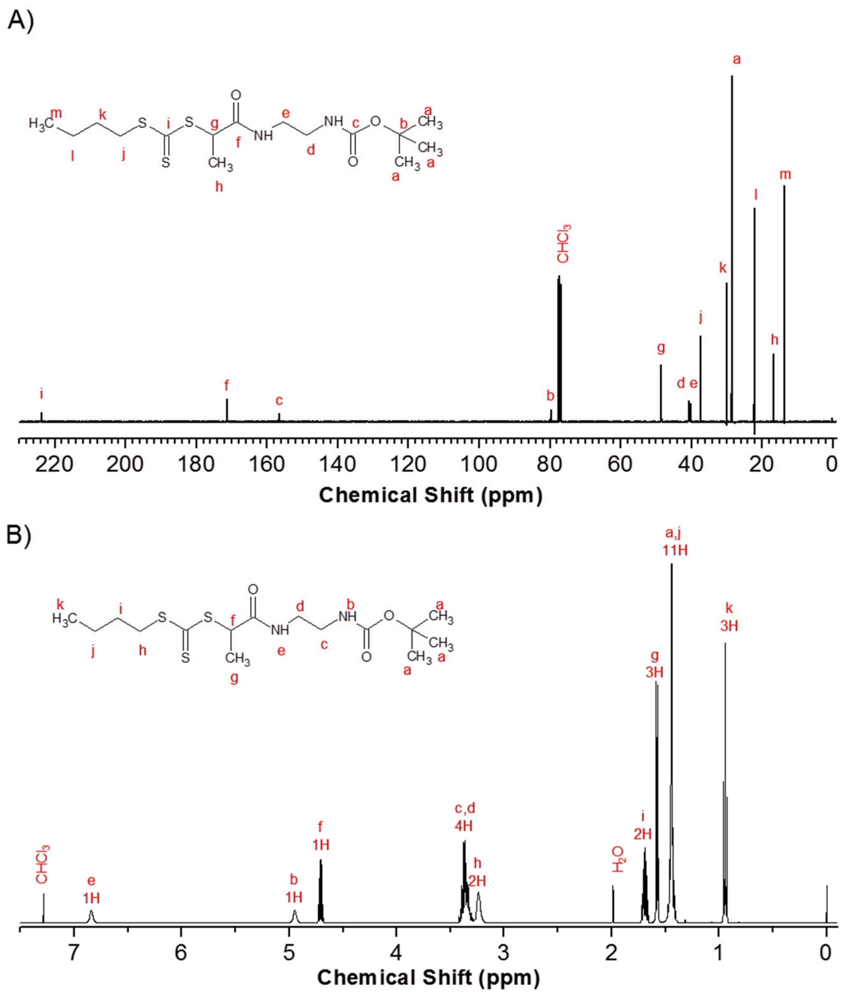

Figure S3. ^{13}C-NMR (A) and ^{1}H-NMR (B) spectrum of the N-boc-ethylenediamine C_4-RAFT agent recorded in CD_3Cl (500 Hz).

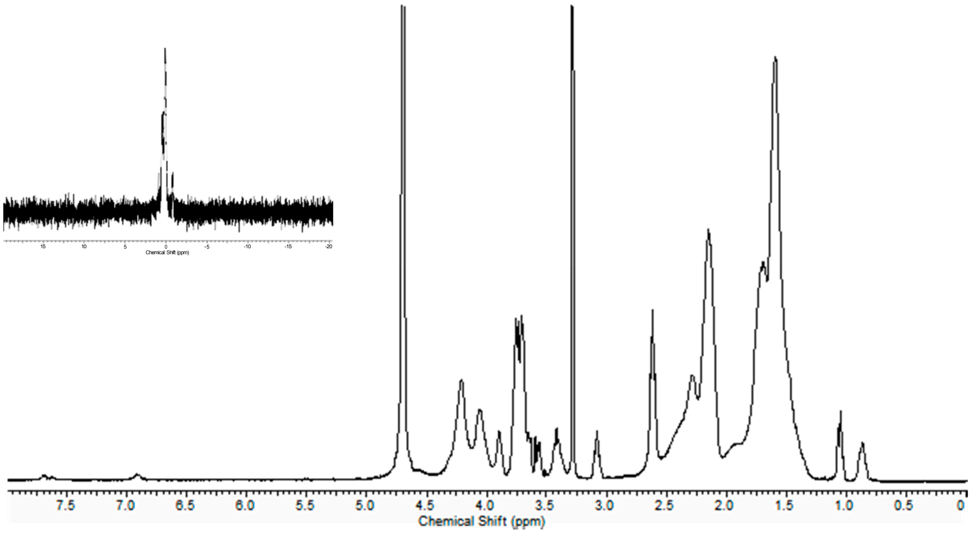

Figure S4. ^{1}H-NMR and ^{31}P-NMR (inset) spectra of RAFT-MAEP$_{10}$-AAm$_{20}$-NH$_2$ recorded in D_2O (300 Hz).

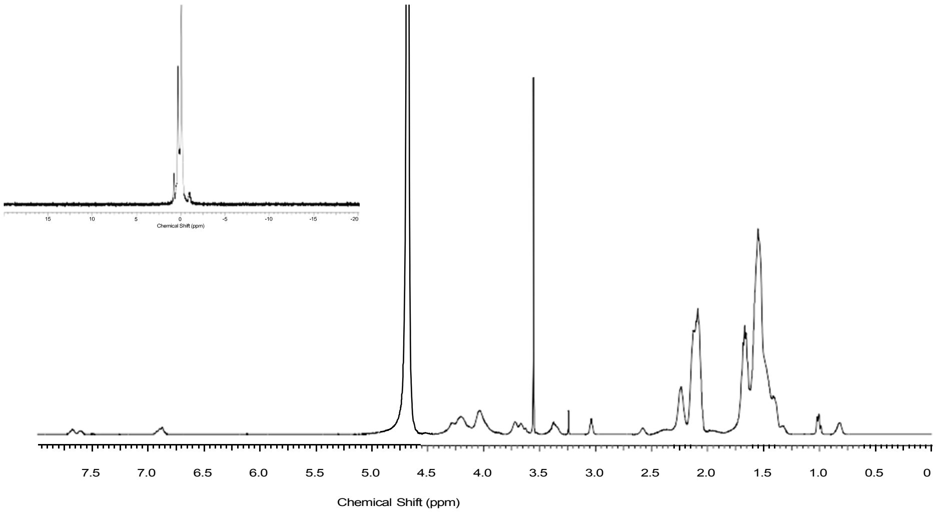

Figure S5. ^{1}H-NMR and ^{31}P-NMR (inset) spectra of RAFT-MAEP$_{10}$-AAm$_{60}$-NH$_2$ recorded in D$_2$O (300 Hz).

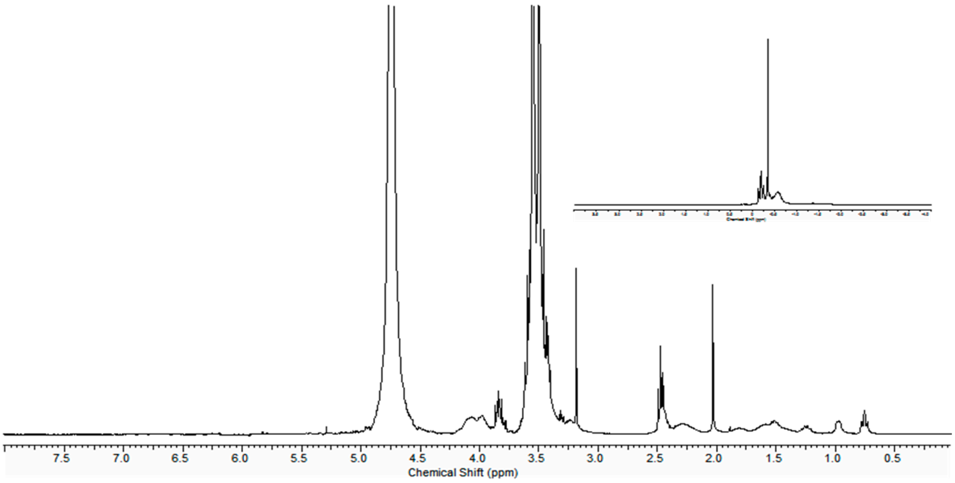

Figure S6. ^{1}H-NMR and ^{31}P-NMR (inset) spectra of RAFT-MAEP$_{10}$-MPEG recorded in D$_2$O (300 Hz).

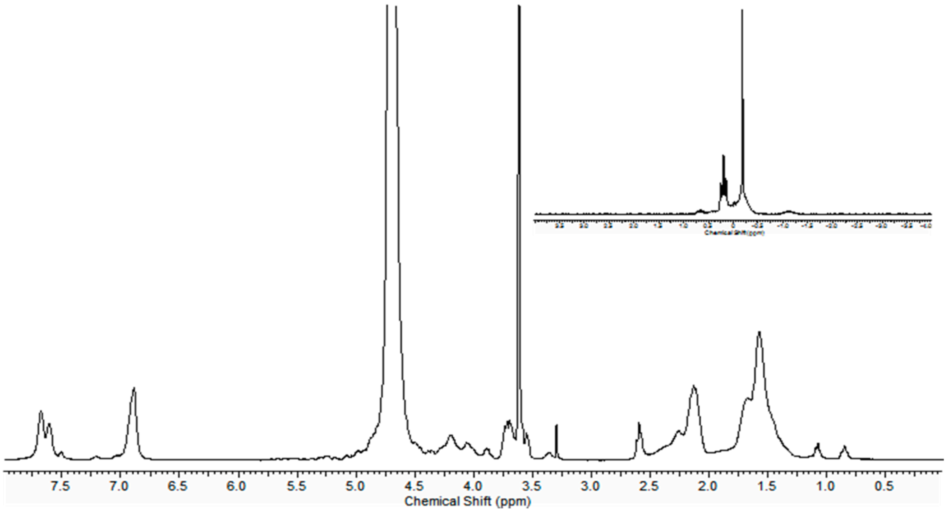

Figure S7. ^{1}H-NMR and ^{31}P-NMR (inset) spectra of RAFT-MAEP$_{10}$-AAm$_{40}$-MPEG recorded in D$_2$O (300 Hz).
